# Alexithymia, Suicidal Ideation, and Self-Esteem As Psychological Factors Affecting Chronic Kidney Disease Patients Under Haemodialysis: A Contextual Review

**DOI:** 10.7759/cureus.54383

**Published:** 2024-02-17

**Authors:** K Ramya, D Jagadeswaran

**Affiliations:** 1 Clinical Psychology, Saveetha College of Allied Health Sciences, Saveetha Institute of Medical and Technical Sciences, Saveetha University, Chennai, IND; 2 Renal Science and Dialysis Technology, Saveetha College of Allied Health Sciences, Saveetha Institute of Medical and Technical Sciences, Saveetha University, Chennai, IND

**Keywords:** self-esteem, suicide risk, depression in chronic illness, emotional factors, suicide ideation, socioeconomic impact, alexithymia, psychological effects, dialysis challenges, chronic kidney disease

## Abstract

Chronic kidney disease is a universal topic gravitating towards various aspects of widespread illness, impacting the overall well-being of human beings. Patients with longstanding renal complaints under dialysis encounter challenges correlated with physical, intuitive, and socio-economic conditions to a greater extent in their daily existence. These portions may include changes in the appearance of a person, restricted physique movements, curbed diet, duration of surgical protocols, travelling time during the period of prevention, financial load, role reversal in the family followed by ruining their livelihood, deprived social rank, difficulty in relational, cordial relationships, and so on. Excluding these details, the sick may be profoundly influenced by sorrow, health anxiety, despair, itching, the impoverished essence of vitality, dysfunction in sexual intimacy, impaired cognition, disturbances in disposition, sleeping fluctuations, frequent panic attacks, delirium, brain-afflicted degeneration disabilities, etcetera. Our analysis focuses on exploring a few unidentified intrinsic factors that distinguish these views over combined elements due to the existing disorder.

## Introduction and background

Chronic kidney failure (CKD) is an irreversible, progressive decline where the body ceases to maintain the balance of metabolism, including anaemia, uraemia, electrolyte instability, and metabolic acidosis, which might lead to endocrine abnormalities [1-3]. Haemodialysis is one of the most accepted treatment methods for CKD. It is widely believed that the concurrent existence of deterring properties and co-existing altercations leads to unfortunate consequences for one’s welfare. Therefore, these situations may require therapeutic interventions [4,5].

In contemporary times, wellness professionals need to understand the true connection with the internal state of consciousness, which contributes to alleviating illness. While multiple papers are being published and comprehensive research studies have been ongoing for over two decades in psychology, consolidating the psychological factors related to haemodialysis remains unfeasible for the investigator at this point. Moreover, the observable information regarding the same may vary. As a researcher, one may discover many motives behind such penurious scenarios. For example, it is feasible to contend that a clinically observed study with captivating results might be limited to small-scale samples. In such instances, it may be demanding to evaluate, replicate, and report the progress of the study’s findings.

Another constraint in the dialysis research area is that a substantial number of mental health experts shift to professions with fewer challenges and less tiresome duties [6]. Hence, we are positioned in the indeterminate sphere of the constrained knowledge base about the many barriers linked with haemodialysis.

Exploring the psychological factors among haemodialysis patients was performed by methodically searching subsequent databases, including Google Scholar, PubMed, and ProQuest. The main terms, such as haemodialysis, dialysis, chronic kidney disease, psychological factors, psychological problems, alexithymia, suicidal ideation, self-esteem, and quality of life, were used to gather relevant articles. Databases were utilised both individually and collectively, including these combinations of fundamental terms. The references in the lists mentioned above were carefully scrutinised to eliminate redundancy. We have specifically selected English-language articles incorporating data on haemodialysis patients with CKD.

## Review

Understanding alexithymia through the neurosis model

Despite the absence of limitations on the search timeline and research design and initially perceiving the groundwork as minuscule, the multidimensional nature of alexithymia has consistently intrigued researchers to explore its associations with human intellect over the decades. The label is derived from the primitive Greek colloquial ‘a, lexis, and thymos’, denoting a lack of words for emotions. It carries different attributions that may induce struggles in identifying sentiments, an inability to describe feelings to another, sensing internally, and possessing narrowed speculative sufficiency. Equally, the sufferer may have hindrances in improvised processing that may prompt minimal verbalism and recall [7]. In this context, the patient on a dialyser may jeopardise trauma from the incision to the post-procedure, instigating a sense of suppressed sensation and thereby manifesting uncertainty in the present. Also, the future might be engulfed by despair [8].

Alexithymia is a psychosomatic element narrating the psyche’s opinion and peculiarities that may influence human contentment. In the recent era of scientific consideration, especially on the psychological front, few insightful theories illustrate the association enclosed by reactions, attributes, and outcomes on the built [9,10]. The neurosis paradigm, as a schema, describes the progression of alexithymia. While not in alignment with this model, alexithymia stems from the unsettled emotional state of the self. This evolution occurs during the growing phase of the child and the social environment in which they grow up. The framework also suggests that the intrapsychic disharmony that outlines the patient's exacerbating spontaneous arousal upsets the temperament. Further, probing into an unconscious state of presuppositions denotes that there is always an interconnection between the anatomy, attitude, and temper that keeps evolving throughout the lifespan.

Origin and root causes of alexithymia

The origin of the subconscious replica is linked to various conflicts within the inherent structure of mental composition and external pressures. In 1952, a well-known psychologist, Karen Horney, investigated the concept that demeanour is intricately connected to deficits in selfhood [11]. Consecutively, an American psychiatrist, John Nemiah, and his co-worker, Peter S, plotted the analytical patterns of disarrayed individuals who apprehend tension-urged hurdles. They conclude that the convalescents were comprehending great interference in conveying convictions while going through soreness; thus, the phrase means ‘no cues of how they feel’. The aetiology may result from a loss of apperception from childhood unfolding into adulthood and is divided into primary and secondary. The former occurs from constant edginess from a hostile caregiver during the childhood phase or adolescence, while the latter is rooted in never-ending stress because of ‘normal’ stressors in grown-up life [12]. Supplementary markers are scantiness in automatic empathy and intents of fantasy. Fundamentally, the predicament is grounded in the unconscious scope of discipline that characterises the link mediating bodily sensations and the individual’s histrionic aspect. Patients with alexithymia may have dilemmas in accepting basic happiness or sadness, both at the surface and in intensity. The common trait intervening in autism and disrupted awareness is the absence of sensitivity in the patients. Since they cannot feel their standpoint, it seems arduous to feel togetherness in general [13,14].

Prevalence of alexithymia in haemodialysis patients

In the modern-day scenario, literary work may be prevalent in the bedlam of dynamism, conversion, apprehension, and somatoform. A review conducted on the Native populace of Greece concludes that 52% of the dialysed causality was dispensed to impassiveness, while 18% had possibilities of the same [15]. Botheration in reactivity occurs as an independent risk factor or with additional comorbidities. In the past, the published work on the same was way limited, as the ratios were calculated to be 8.9%, 8.3%, and 4.7%, respectively. Hence, its prevalence in general society may be less than 10%.

Indicators of alexithymia in haemodialysis patients

The damaging unsoundness described may give rise to biological and irrational derangements in the prospects surrounding the concerns of the affected individuals and their families, posing a fatal risk for both the subjects and their caregivers [16,17]. This state, characterised by cognitive and affective deficits, encompasses experiences such as outrage, confusion [18], disappointment, an inability to read appearances, a racing heart, rapid palpitations, jitters, soreness, desensitisation, and emptiness from a farther point based on views from which they would not be able to feel inward or relate to objective responses [19-21]. Maintenance dialysis patients may be affected by severe discomfort, general desolation, the sedentary standard of comfort, etcetera, in which the judgement adapts to a coping mechanism where those channels in the direction of the cerebrum are latched, which incumbents an apathetic, intermittent effect. Dialysing sufferers exhibit heightened nervousness related to diagnostic exposure regularly. They may also bear fatigue beyond hospital hours [22,23]. They may experience different effects such as wild impulses, an inability to figure out and express the sense behind perspective, establishing alternatives, flight-fright responses owing to touch, noises, sight, an incapacity to mark what they feel, a strange, unfamiliar nature close to their household, peers, or sudden outbursts. In connection with recurring pathologies, it was observed that hypnosis works to relieve afflictions to a nominal degree [24].

Interventional strategies for alexithymia

In terms of intervention, it is crucial to evaluate alexithymia with commonly used questionnaires such as the Toronto Alexithymia Scale and Perth Alexithymia Questionnaire, which are presented in the references part of this paper [25]. Following the clinical assessment, the next step for the therapist may be to envision alexithymia as an aspect of a lack of emotional awareness and regulation. Holistically, this involves introducing psychotherapy in the form of empathetic talking therapy, expressive writing, and creating art to ease the difficulty of identifying and expressing emotions. It is also essential to conduct therapies such as sports, relaxation, body movement, and dance to enhance body awareness. Moreover, group therapy in terms of psychodrama and role plays can help them add meaning to their vocabulary. A focused, insight-oriented approach based on a one-on-one mode can help effectively describe how they feel about themselves and be in touch with their emotions. Other interventional strategies may include psychotherapy, which helps identify and express their emotions effectively [26].

Suicide ideation

Definition and Primary Rate

Suicidal ideation is mostly known as contemplation or imagination, explaining the extent of wishes, obsessive thoughts about the demise or killing oneself, and instant plans to perish or death ideas referred to as disjoined impressions of annihilation [27-29]. Such notions can occur in depressed and non-depressed individuals, while tiring agony and terminal sickness can create rumination in the latter case. Concerning critical conditions, there is a significant association interpolated among nephritic malfunction, depression, and judgement in general [30,31]. The proportion of the community dying for the above reason can be permutated based on age, class, race, gender, and extra statistical units [32]. A survey of the Southeast Asian population shows that the predominance of lethal thought is around 9% globally, which is relevant to the highest numbers in Europe. It has also been investigated that its prevalence in maintenance dialysis is 47% higher than in non-dialysis natives [33]. Assessing on-dialysate patients for factors such as uremic toxins on the side of stratum supremacy may be crucial in accentuating the significance of harmful thoughts [34].

Theoretical perspectives on suicide ideation

In the research field of suicide, three hypotheses emerge: the interpersonal theory of suicide, the integrated motivational-volitional model, and the three-step theory. These ideologies emphasise how suicidal ideation progresses and might later develop into an actual suicidal attempt [35,36]. The interpersonal approach consists of four core constructs: thwarted belongingness, perceived burden, hopelessness, and acquired capability. Thwarted belongingness may be rooted in disconnecting from society and the state of being a part of the family, friends, workplace, or any acquaintances. Later, in sensed overloading, individuals might have muddled thinking that their death could be more of a reward to others than being alive. While hopelessness is fairly understood, the adopted forte is usually one’s ability to kill oneself against the basic biological instinct of survival. It usually results from life stressor events such as physical abuse, a history of suicidal behaviour, war exposure, etc. The proponent postulates that an overpowering drive to be related to someone and anticipated feelings of being an obstruction to others might be the proximal indicators of passive ideation when coupled with hopelessness and a learned ability to attempt suicide, which might later grow into an active imagination that may lead to fatal or non-fatal behaviour [37].

The integrated motivational-volitional inference is a tripartite factor-based concept that describes the suicidal ideation that emerges initially in the pre-motivational phase of the ‘abnormal’ state of the body or mind, alongside the environment and life stressors of the being. Scaling towards the motivational phase, there are factors such as being downtrodden, feeling undignified, and so on that might help in shaping the passive ideation into a real thought. Further, active ideation is triggered in the volitional phase, where the individual finds means or possibility to enact due to a violent temper, physical pain sensitivity, or copycat suicidal patterns [38].

The three-step theorem hypothesises that suicide ideation originates from ordeals and pain as an outcome of conditioning. The second step imposes a state of ruptured connectedness to society while overweighing the pain, leading to mild to moderate ideations. In conjunction with an interpersonal proposal, the third step incorporates acquired capability, genetic conditions, and practical means of finding fatal ways to attempt [39].

Recognising suicide ideation in haemodialysis patients

Signs and Symptoms

When considering generic phenomenology related to pondering over dying in dialysis patients, manifestations include hopelessness, helplessness, sleeplessness [40], deficiency in worth and replica, inherent neglect, insufficient support, episodes of melancholy [41], unworthiness, prevailing to be a burden to the populace around them, contrary perceptions, standing anguishes, malicious assumptions, etc. [42]. In the case of dialyser rehabilitation, these can be specified as bleak symptoms and extreme misery through hemofiltration. Individuals who reflect on the same may reveal marked transformations in their usual routine, preoccupied beliefs about passing away, willingness to die, recklessness, and isolation, cyclical mood swings, excessive use of drugs, substances, or alcohol, abrupt distribution of wealth elsewhere, risky behaviour, and serious transitions in temperament. Enduring dialysis may sustain acute psychical aggravation such as fear, restlessness [43], pessimism, permanent worries, and doubts triggering non-assertiveness [44,45]. Outpatients may not be enthusiastic in their personal and respective lives because of bedridden movements and solid tiredness [46]. These might be fronting dyadic unlikeness, frustration, guilt, and reduced chronic care turning to treachery. Scenarios such as non-prescription medicinal abuse and hysterical mayhem might increase the probability of dangerous speculation [47]. Therefore, formulating suicide prevention protocols is mandatory in the mental health sector of haemodialysis care.

Long-term strategies for suicide prevention and mental wellness in dialysis patients

An action plan, such as the collaborative care proposed by the World Health Organization, may be useful in preventing suicidal risk among the maintenance dialysis crowd. The process coordinates both mental health practitioners and nephrologists to understand the underlying mechanisms of pain and trauma, which may help in addressing depressive symptoms and intervening later on [48].

Certain coping skills, specifically those based on activities and sensibility in general, may be implemented as part of the regimen to reduce suicidal ideation and enhance patients' overall welfare. Engaging with a favourite pastime, socialising with the public, being good to themselves, talking to a crisis counsellor, calming themselves, and sitting with their feelings until they slowly pass away can enable them to lead a conscious life amidst the hospital hours and homestay.

Other techniques, such as establishing a crisis helpline, restraining access to common means of suicide such as pesticides, creating awareness about suicide prevention in social media, life skills training for medical and mental health practitioners, earlier clinical assessments, and administering follow-ups, might decrease the risk of suicidal ideation among haemodialysis patients [49].

Understanding self-esteem

Definition and Importance

In the domain of scientific surveys, approving one's status plays a vital part in the intellectual and advancing field of civil literacy. Unique assurance plays a central part in every single person’s welfare in society. In 1962, the term self-esteem was coined, pointing out the favourable evaluation of a whole being. He also mentions that a higher degree of reverence for existence may lead to autonomic assurance, vehemence, capability, invulnerability, and coherence. Various integral sections contribute to turmoil in childhood, inclination to ailment, fiscal tribulation, social seclusion, demographic factors, and gender dysphoria that may influence a person’s overall character [50].

The autogenic value of maintenance dialysis lies in confronting periodic threats from the medical regime, alterations in the torso, and locus of control. Autonomy is important in accepting the current health situation and being unwilling to submit to unhealthiness. Therefore, it is obvious that it has an impact on positive and negative outcomes, which is relevant in providing remedial support calculated based on the mortality rate [51].

Signs of low self-esteem in haemodialysis patients

The dialysis casualties may indicate lower esteem inhibited by various factors, such as abandonment issues, itching leading to patchy skin [52], sexual dysfunction [53], shortcomings in behaviour, sleep deprivation [54], unemployment problems, an inability to efficiently communicate, challenges in navigating the real way of progressing against pensive turbulence and forthcoming worries, a lack of control over things, and struggles in civil and personal accord, among others [55]. In addition to these signs, excessive water consumption may lead to bloating, which, in turn, might disturb subjective consciousness adversely. Assessing self-esteem has become a necessary task in examining the integral dialysis inmates. Considering the extended period of medical administration and trials, patients may have less confidence, which can directly weaken the coherence of medication [56].

There are ever-changing dynamics that can affect day-to-day activities and performance levels in families and society [57]. As a result, the standard of comfort and behaviour is drastically altered, and they may also assess themselves less favourably compared to healthy residents [58]. Due to the malfunctioning of the patient’s stamina, families may long for an exchange of accountability, leading to unfulfilled needs and overall dissatisfaction. Subsequently, core needs such as joy, communal awareness, freedom, strength, and group dignity are influenced. In contrast, they feel that their responses are not acknowledged and validated but, instead, are distinctively rejected [59]. To boost the confidence of the dialysis population, it is essential to impart knowledge and awareness about their illness.

Promoting self-care and self-compassion

The collaborative healthcare team of nurses, nephrologists, and therapists may devise a holistic program comprising mindfulness, deep relaxation techniques, and spiritual practices, teaching them how and when to ask for mental health support when required. The patient’s active involvement plays a vital role in enhancing their well-being. These may encompass routinely self-assessing their symptoms, participating in decisions, regulating their negative emotions such as trepidation and plight, following a healthy diet and engaging in physical activities, and accepting their illness as a part of their own lives [60,61].

Understanding their health condition and their ability to use the available medical resources effectively may reduce the symptom burden while slowing down the progression of the disease. It may kindle confidence to overcome the strenuous phase of cure and compliance.

## Conclusions

Patients who undergo dialysis harbour prejudiced views concerning survival due to excruciating aches. This adverse rumination may advance to severe desperation, causing them to mull over thoughts of self-harm and end their lives rather than succumbing to the ailment. Moroseness is attributed to lethargy and reduced endurance. Symptomatic portrayal may occur collectively or as a separate part. Based on a prognosis, our inquiry aims to delve into alexithymia, suicide speculation, and egotism in patients subjected to haemodialysis. Employing appropriate interventions to promote the patient’s overall well-being while addressing dialysis care is essential as well. Likewise, psychological counselling may provide therapeutic space to express their feelings occasionally.
